# Willingness of patients with cancer pain to participate in end-of-life decisions: a multi-center cross-sectional study from three coastal provinces in southern China

**DOI:** 10.1186/s12904-022-01108-x

**Published:** 2022-11-26

**Authors:** Xi Ke, Hongyu Zhu, Yu Zhang, Ling Yang, Simei Shi, Fang Zhu, Huiyu Luo

**Affiliations:** 1 Department of Abdominal Oncology, Clinical Oncology School of Fujian Medical University, Fujian Cancer Hospital, Fuma Road 420#, Fuzhou, 350014 China; 2 Department of Internal Medicine-Oncology, Shaanxi Cancer Hospital, Xian, 710061 China; 3 Department of Gastroenterology-Oncology, Cancer Hospital Affiliated to Guangxi Medical University, Nanning, 530000 China; 4 Department of Gastroenterology-Oncology, Sun Yat-Sen University Cancer Prevention and Treatment Center, Sun Yat-sen University Cancer Center, Guangzhou, 510060 China; 5 Department of thoracic oncology, Clinical Oncology School of Fujian Medical University, Fujian Cancer Hospital, Fuzhou, 350014 China; 6 Nursing management department, Clinical Oncology School of Fujian Medical University, Fujian Cancer Hospital, Fuma Road 420#, Fuzhou, 350014 China

**Keywords:** Cancer pain, End-of-life decisions, Questionnaire, Cross-sectional study

## Abstract

**Background:**

Little is known about patients’ intention for participation in end-of-life decisions (EOLD) in three coastal provinces in southern China. This study aimed to explore the willingness of patients with cancer pain to participate in EOLD and potential influencing factors.

**Methods:**

A multi-center cross-sectional study was performed in three coastal provinces in southern China. Two hundred and thirty patients with cancer pain were recruited and consented to fill out the questionnaires. The patients’ willingness to participate in EOLD, demographic and disease-related data was surveyed.

**Results:**

In total, 223 patients completed and returned the survey (response rate = 96.95%). One hundred four cases (46.64%) were willing to participate in EOLD. 119 (54.36%) cases not willing to participate in EOLD, respectively. Multivariate logistic regression analysis shows that educational level (OR: 0.683, 95% CI: 0.482–0.966), history of alcoholism (OR: 8.353, 95%CI: 2.535–27.525), Eastern Cooperative Oncology Group (ECOG) score (OR: 0.645, 95% CI: 0.450–0.925) and experience of explosive pain (OR: 6.367, 95% CI: 3.103–13.062) and clinical rescue (OR: 3.844, 95% CI: 1.722–8.577) had significant effects on EOLD intention (*P* <  0.05). Finally, a predictive model combined above five factors was established, which showed a good discrimination (area under receiver operating characteristic curve: 0.849, 95% CI: 0.796–0.899, *P* <  0.001) and calibration (Hosmer-Lemeshow Test: Chi-square = 10.103, *P* = 0.258) for which patients more willing to participate in EOLD.

**Conclusions:**

The willingness of patients with cancer pain to participate in EOLD is at a modest level in three coastal provinces in southern China. Patients with lower educational level, history of alcoholism, better health status and experience of explosive pain and clinical rescue may be more prone to participate in EOLD.

**Supplementary Information:**

The online version contains supplementary material available at 10.1186/s12904-022-01108-x.

## Background

Cancer-related pain, reported by more than 70% of patients, is one of the most common and troublesome symptoms, which can occur at different stages of cancer. Despite the continuous optimization of analgesic treatment strategies, cancer-related pain may be inadequately controlled in up to 50% of patients [1]. Pain doesn’t only devastate the quality of life (QOL) of patients with cancer, but also are regarded as a sign of tumor recurrence, metastasis, and treatment ineffectiveness by patients and their families [2]. Patients with cancer pain are often in a critical state of fear of death due to the incurable nature of cancer. They are more vulnerable to anxiety, depression, fatigue, and lack of a positive outlook. When the status of an illness continues to deteriorate, the majority of patients begin to think about the meaning and purposes of life and issues around death [3]. Thus, understanding patients’ wishes, values and expected goals is of great significance for formulating patient-centered end-of-life (EOL) care and improving the quality of life in the EOL stage.

End-of-life decisions (EOLD) (including advance directives (ADs), do not resuscitate/do not intubate, last wishes and so on) are unavoidable topics that all palliative care physicians, patients, and their caregivers must face [4]. In the Western society and some developed Eastern countries like Japan, Korean, and Singapore, increasing patients participated in EOLD since the passage of the Patient Self-Determination Act (USA) in 1991 [5–10]. They tend to express their thoughts on dying decisions and death in writing, and to believe that this practice is very necessary [8, 9]. The doctors and nurses of medical institutions pay also more and more attention to patients’ autonomy and decision-making power [11]. Previous studies have demonstrated that quality EOL care can be achieved when patients receive the treatments they desire [12, 13].

Although some studies focused on the preference of EOL care and cancer patients’ and caregivers’ decision-making practices in China mainland [14–16], little was known about the true thoughts of patients with cancer pain to participate in EOLD. The aim of this study was to investigate the willingness of such patients to participate in EOLD, preference of EOL care and associated factors in three coastal provinces in southern China.

## Methods

### Design and setting

This study was a cross-sectional study, which carried out in three tertiary cancer hospitals (Fujian Cancer hospital in Fuzhou, the affiliated cancer hospital of Zhongshan University in Guangzhou and the affiliated cancer hospital of Guangxi University in Nanning) in Southern China because the tertiary cancer hospital is a provincial cancer treatment center, which can accept cancer patients from all regions of the province (including rural and urban areas). This study was reviewed and approved by the centralized ethics committee of Fujian Cancer hospital (SQ2018–039-01). The data collection was completed between August 2020 and December 2020.

### Participants

Potential eligible in-hospital patients with cancer pain were screened according to the following inclusion and exclusion criteria. The inclusion criteria were: (1) age ≥ 18 years old, with good language communication skills; (2) patients with malignant tumor diagnosed by histology or pathology; (3) NRS (numerical rating scale, NRS) ≥ 1 point during treatment; (4) mental stability; (5) voluntarily participate in this study under the principle of informed consent. The exclusion criteria were: (1) not answer or not fill in the questionnaire, (2) those patients who have unstable vital signs. The questionnaire used in this study contained 20 items. Ten participants per item were adopted to guide the sample size calculation according to an epidemiological method previously reported [17]. The estimated sample size would be 200. Taking into account a projected 85% completion rate, the total sample size was 230 in this study.

### Measures

#### Questionnaire

The questionnaire of willingness to participate in EOLD was designed based on consulting relevant literature [18]. The questionnaire consists of four parts (the supplementary file 1): (1) demographic data: gender, age, educational level, religious belief, marital status, residence and monthly family income; (2) Disease-related data: disease diagnosis, cancer stage, history of smoking and alcoholism, etc.; (3) Pain score (NRS, number rating scales), Eastern Cooperative Oncology Group (ECOG) score at the time of investigation, and Nutritional risk screening (NRS 2002); (4) Willingness to participate in EOLD (the core part of the questionnaire): there are 1 decision item and 3 items about influencing factors, which are: ① whether you are willing to participate in EOLD (yes, no); ② Influencing factor 1: have you experienced explosive pain (yes, no); ③Influencing factors 2: causes of pain outbreak (disease deterioration, Improper use of analgesics, others); ④ Influencing factor 3: have you experienced clinical rescue (yes, no). For those patients willing to participate in EOLD, death related topics were further surveyed, including preference to EOL decision-maker, preference to life-support care and preferred place of death.

#### Procedure

Before performing this investigation, data collectors were trained on what EOLD is and how to interpret the purpose, process and potential benefits / risks to the participants to ensure the quality of data collected. Five trained collectors screened the eligible patients from the in-hospital patients with cancer pain according to the inclusion and exclusion criteria. Then, the data collectors would explain the definition of EOLD and relevant survey information to the potential participants and invited them to participate in the current study. After the patients fully understood the meaning of the questionnaire and agreed to participate in this study, they were required to sign a written informed consent. For those patients who have difficulty in writing, the patient’s family signed the written informed consent on behalf of them. After obtaining the informed consent from each participant, the data collectors guided the patients to complete the questionnaire in the form of face-to-face interviews.

### Statistical analysis

The normal distribution of outcome variables was confirmed using the Kolmogorov–Smirnov test. Categorical variables were expressed as percentages and compared with the Chi-Square test. Continuous variables were presented as mean and standard deviation, and compared by using the independent-sample Test or Mann–Whitney U test. 95% confidence intervals (95% CI) were calculated for the median length. First, univariate logistic regression was used to measure the independent relationships between willingness to participate in EOLD and each predicting variable. Then, factors with a *P* value < 0.1 in univariate analysis were further included in multivariate logistic regression model and analyzed using a backwards model selection procedure (elimination criterion: *p* > 0.05) [19]. Finally, those factors with a *P* value < 0.05 that were utilized to build the final predictive model. The receiver operating characteristic curve (ROC), calibration plot, and Hosmer-Lemeshow test were used to validate the discrimination and calibration of the model. Statistical tests were performed using the SPSS version 22.0 (IBM Corp., Chicago, USA). All *P* values were two-tailed, and statistical significance was set at *P* <  0.05.

## Results

### Basic information of participants

The basic information of patients was shown in Table 1. A total of 230 patients with cancer pain participated in the study, 7 patients did not complete the questionnaire. Finally, 223 (response rate 96.9%) patients were included in the final study analysis. Of which, 115 participants (51.6%) were from Cancer hospital of Fujian province, 73 participants (32.7%) from the affiliated cancer hospital of Zhongshan University in Guangzhou, and 35 participants (15.7%) from the affiliated cancer hospital of Guangxi University in Nanning, respectively. 138 (61.9%) were male and 85 (38.1%) were female. The mean age of the patients was 55.8 ± 14.1 years and the body mass index (BMI) was 20.9 ± 3.3. Two hundred twelve patients (95.1%) were married, 86 (38.6%) patients from rural areas, and 110 (49.3%) patients no religious belief. The majority of patients had a relative low-level education experience with a proportion of 70.4% (39.5% for primary school and below and 30.9% for middle school, respectively). The rate of previous smoking and alcoholism were 30.94 and 12.1%, respectively. About half of the patients (57.4%) have low house-held income. In terms of clinical disease characteristics, 64.1% of patients were diagnosed as gastrointestinal tumors, 22.86% as respiratory tumors, 4.5% as urinary tumor, 1.3% as gynecological tumor, and 7.2% as others, respectively. The rate of patients with pathological stage III or above accounted for 92% (in total, 205 cases). On the whole, the nutritional status and general physical condition of the patients included are still acceptable. The nutritional risk score 2002 (NRS2002) and the ECOG score were 2.1 ± 1.1 points and 1.4 ± 1.0 points, respectively. In terms of the pain severity，the average NRS (numerical rating scale) score was 2.3 ± 1.0 points. About three-quarters of patients (74.5%) received a three-step analgesic treatment. 126 (56.5%) patients had an experience of explosive pain. The main causes included disease deterioration (43.9%) and improper use of analgesics (30.9%). Daily dose of opioid analogues used in the patients was 117.9 ± 115.8 microgram. Finally, only 57 (25.6%) patients have ever experienced a clinical rescue.

**Table 1:** Participants’ baseline characteristics (*n* = 223)

Variables	Total (*n* = 223)	Willing to participate in EOLD		*P* value
		Willing (*n* = 104)	Unwilling* (*n* = 119)	
Age, ys	55.8 ± 14.1	58.9 ± 13.3	53.1 ± 14.2	0.002
Gender, n (%)				0.514
Male	138 (61.9)	62 (59.6)	76 (71.1)	
Female	85 (38.1)	42 (40.4)	43 (28.9)	
Body mass idex	20.9 ± 3.3	20.9 ± 2.5	20.9 ± 3.8	0.881
Marital status, n (%)				0.638
Married	212 (95.1)	98 (94.2)	114 (97.8)	
Divorced/widowed	7 (3.1)	4 (3.9)	2 (0.0)	
Unmarried	4 (1.8)	2 (1.9)	3 (2.2)	
Education, n (%)				0.020
Primary school and below	88 (39.5)	48 (46.2)	40 (46.7)	
Middle school	69 (30.9)	35 (33.7)	34 (20.0)	
High school	40 (17.9)	15 (14.4)	25 (20.0)	
Above high school	26 (11.7)	6 (5.7)	20 (13.3)	
Living area, n (%)				0.057
Rural	86 (38.6)	47 (45.2)	39 (33.3)	
Urban	137 (61.4)	57 (54.8)	80 (66.7)	
Religiosity, n (%)				0.248
Yes	113 (50.7)	57 (54.8)	56 (35.6)	
No	110 (49.3)	47 (45.2)	63 (64.4)	
House-held income, n (%)				0.203
Low	128 (57.4)	59 (56.7)	69 (58.0)	
Middle	84 (37.7)	37 (35.6)	47 (39.5)	
High	11 (4.9)	8 (7.7)	3 (2.5)	
Previous smoking, n (%)				0.527
Yes	69 (30.9)	30 (28.8)	39 (32.8)	
No	154 (69.1)	74 (71.2)	80 (67.2)	
Previous alcoholism, n (%)				< 0.001
Yes	27 (12.1)	23 (22.1)	4 (3.4)	
No	196 (87.9)	81 (77.9)	115 (96.6)	
Tumor site, n (%)				0.408
Digestive	143 (64.1)	67 (64.4)	76 (63.9)	
Respiratory	51 (22.9)	27 (26.0)	24 (20.2)	
Urinary	10 (4.5)	4 (3.8)	6 (5.0)	
Gynecological	3 (1.3)	0 (0.0)	3 (2.5)	
Other	16 (7.2)	6 (5.8)	10 (8.4)	
Pathological stage， n (%)				0.785
Stage I	3 (1.3)	2 (1.9)	1 (0.8)	
Stage II	15 (6.7)	8 (7.7)	7 (5.9)	
Stage III	84 (37.7)	37 (35.6)	47 (39.5)	
Stage IV	121 (54.3)	57 (54.8)	64 (53.8)	
Pain score (NRS)	2.3 ± 1.0	2.4 ± 0.9	2.3 ± 1.1	0.784
NRS2002	2.1 ± 1.1	2.1 ± 1.2	2.1 ± 1.1	0.844
ECOG score	1.4 ± 1.0	1.3 ± 0.9	1.6 ± 1.1	0.028
Analgesic scheme, n (%)				0.418
One-step	9 (4.0)	3 (2.9)	6 (5.0)	
Two-step	48 (21.5)	26 (25.0)	22 (18.5)	
Three-step	166 (74.5)	75 (72.1)	91 (76.5)	
Daily dose of opioid analogues, mg	117.9 ± 115.8	138.8 ± 169.5	99.6 ± 140.9	0.008
Experience of explosive pain， n (%)				< 0.001
Yes	126 (56.5)	85 (81.7)	41 (34.5)	
No	97 (43.5)	19 (18.3)	78 (65.5)	
Cause of pain outbreak, n (%)				0.006
Disease deterioration	98 (43.9)	43 (41.4)	55 (46.2)	
Improper use of analgesics	69 (30.9)	25 (24.0)	44 (37.0)	
Other	56 (25.1)	36 (34.6)	20 (16.8)	
Experience of clinical rescue, n (%)				< 0.001
Yes	57 (25.6)	45 (43.3)	12 (10.0)	
No	166 (74.4)	59 (56.7)	107 (90.0)	

*ECOG* Eastern Cooperative Oncology Group, *: The patients of group unwilling were composed of those who refused to and did not sure whether to participate in EOLD

### Difference between patients who willing and unwilling to participate in EOLD

The patients of group unwilling were composed of those who refused to and did not sure whether to participate in EOLD. Finally, 104 patients (46.6%) were willing (group willing) and 119 patients (53.4%) unwilling (group unwilling) to participate in EOLD, respectively. The detail comparison was showed in Table 1. There were no differences in gender, BMI, marital status, religious brief, house-held income, previous smoking, tumor site, pathological stage, NRS score, NRS 2002 score, and analgesic scheme (all *P* > 0.05). The patients in the group willing were older than the group unwilling (mean age: 58.9 ± 13.3 VS. 53.1 ± 14.2, *P* = 0.002). More patients in the group unwilling have ever accepted a relative higher-level education with a proportion of 33.3% for high school or above (vs. 20.1% in the group willing, *P* = 0.020). However, more patients in the group willing had a history of alcoholism when compared with the group unwilling (22.1% vs. 3.4%, *P* <  0.001). The ECOG score of the patients in the group willing was less than that of the group unwilling (1.3 ± 0.9 vs. 1.6 ± 1.1, *P* = 0.028). In terms of pain, 81.7% of patients in the group willing have ever experienced an explosive pain, which was significantly more than the group unwilling (vs. 34.5%, *P* <  0.001). The daily dose of opioid analogues of patients who were willing to participate in EOLD was significantly higher than that of group unwilling (138.8 ± 169.5 mg vs. 99.6 ± 140.9 mg, *P* = 0.008). The proportion of pain in the group willing due to disease deterioration, improper use of analgesics and others was 41.4, 24.0 and 34.6%, respectively. Conversely, the proportion of pain in the group willing due to disease deterioration, improper use of analgesics and others was 46.2, 37.0 and 16.8%, respectively. There was significant difference between two groups (*P* = 0.006). In addition, in the group willing, there were more patients had an experience of clinical rescue when compared with the group unwilling (43.3% vs. 10.0%, *P* <  0.001).

### Influencing factors and predictive value on willingness to participate in EOLD in patients with cancer pain

**Fig. 1:**
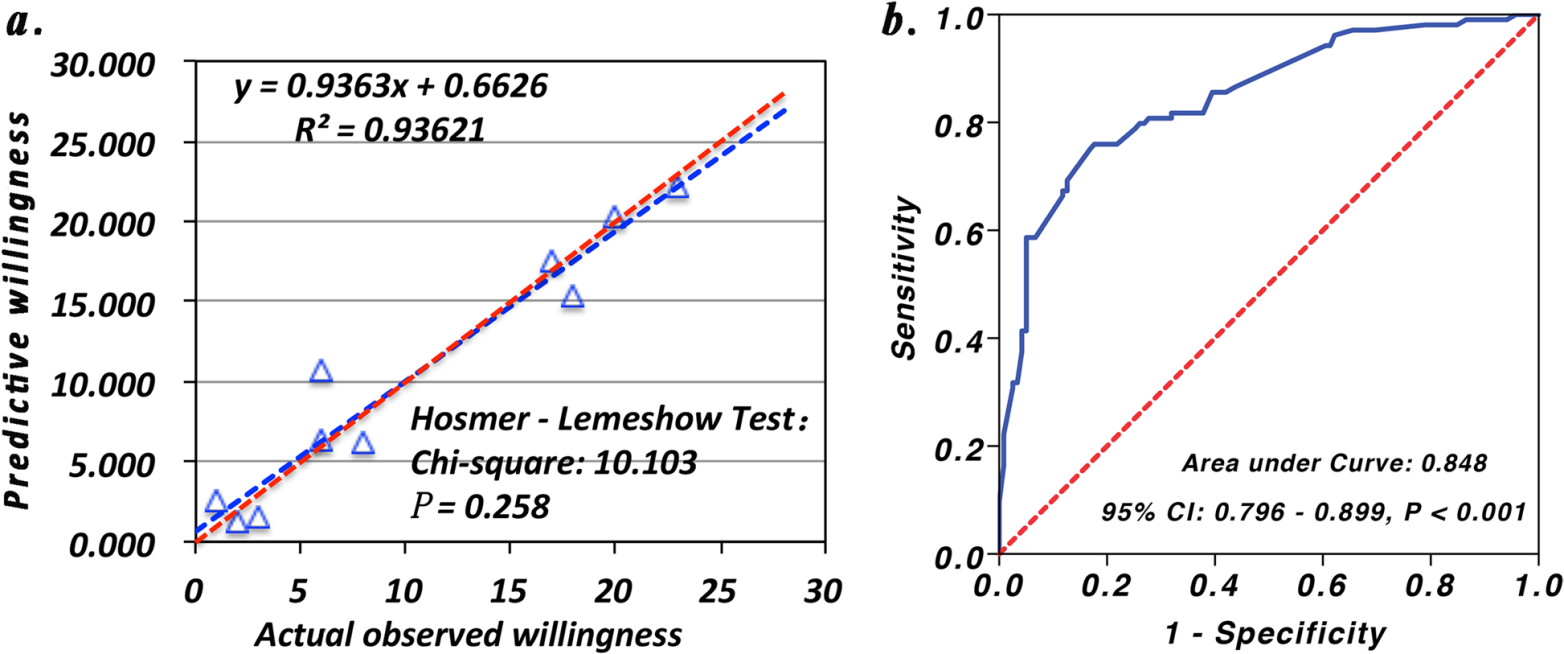
Calibration and prediction ability analysis of a model incorporating five predictors. **a** Calibration curves showed good consistence between the actual observed willingness to participate in end-of-life decisions (EOLD) and the predicted willingness by a predictive model incorporating five predictors including educational level, previous alcoholism, Eastern Cooperative Oncology Group (ECOG) score, experience of explosive pain and experience of clinical rescue (Hosmer-Lemeshow Test: Chi-square = 10.103, *P* = 0.258). **b** The receiver operating characteristic (ROC) analysis for the probability and willingness to participate in EOLD

The results of univariate and multivariate logistic regression analysis were showed in the Table 2. The univariate analysis indicated that seven factors were closely related to whether the patients with cancer pain were wiling to participate in EOLD, including age (OR (95% CI): 1.031 (1.011, 1.052), *P* = 0.002), educational level (0.662 (0.504, 0.870), *P* = 0.003), previous alcoholism (8.16 (2.72, 24.51), *P* <  0.001), ECOG score (0.744 (0.57, 0.972), *P* = 0.03), experience of explosive pain (8.51 (4.557, 15.897), *P* <  0.001), cause of pain outbreak (1.33 (1.051, 1.684), *P* = 0.018), and experience of clinical rescue (6.801 (3.338, 13.856), *P* <  0.001). In addition, the living area (0.591 (0.343, 1.018), *P* = 0.058) and daily dose of opioid analogues (1.002 (1.000, 1.003), *P* = 0.064) had a slight impact on whether the patients are willing to participate in EOLD. After incorporating the above nine indicators with *P* <  0.1, the multivariate analysis showed that only five variables were independent predictors whether the patients with cancer pain are willing to participate in EOLD, including higher educational level (0.683 (0.482, 0.966), *P* = 0.031), previous alcoholism (8.353(2.535, 27.525), *P* <  0.001), lower ECOG score (0.645 (0.450, 0.925), *P* = 0.017), experience of explosive pain (6.367 (3.103, 13.062), *P* <  0.001), and experience of clinical rescue (3.844 (1.722, 8.577), *P* = 0.001). Finally, a predictive model equation containing the above five variables was built as Logit (P) = − 0.431 - 0.382*(educational level) + 2.123*(previous alcoholism) – 0.438*(ECOG score) + 1.851*(experience of explosive) + 1.346*(experience of clinical rescue) (Hosmer-Lemeshow Test: Chi-square = 10.103, *P* = 0.258), and used to calculate the probability that the patients were willing to participate in EOLD. The ROC analysis for the probability that the patients with cancer pain are willing to participate in EOLD showed that the area under curve was 0.849 (95% CI: 0.796–0.899, *P* <  0.001) (Fig. 1).

**Table 2:** Univariate and multivariate logistic regression analysis for willingness to participate in EOLD

Variables	Univariate analysis		Multivariate analysis	
	OR (95% CI)	*P* value	OR (95% CI)	*P* value
Age	1.031 (1.011, 1.052)	0.002		
Educational level	0.662 (0.504, 0.87)	0.003	0.683 (0.482, 0.966)	0.031
Living area (Rural)	0.591 (0.343, 1.018)	0.058		
Religiosity	1.364 (0.805, 2.313)	0.249		
House-held income	1.203 (0.77, 1.879)	0.418		
Previous alcoholism	8.16 (2.72, 24.51)	< 0.001	8.353 (2.535, 27.525)	< 0.001
ECOG score	0.744 (0.570, 0.972)	0.030	0.645 (0.450, 0.925)	0.017
Experience of explosive pain	8.51 1 (4.557, 15.897)	< 0.001	6.367 (3.103, 13.062)	< 0.001
Cause of pain outbreak	1.33 (1.051, 1.684)	0.018		
Daily dose of opioid analogues	1.002 (1.000, 1.003)	0.064		
Experience of clinical rescue	6.801 (3.338, 13.856)	< 0.001	3.844 (1.722, 8.577)	0.001

### Preference of patients with cancer pain to EOL care

Preference to EOL care was further surveyed in 104 participants who are willing to participate in EOLD (Table 3). The EOL decision-maker that participants’ preferred was in the following order: spouse (61.5%), offspring (23.1%), participants’ own (8.7%), parents (2.9%), relatives (2.9%), and friends (1.0%). In terms of preference to life-support care, more than half of the patients are willing to accept medication and nutritional support to extend life (59.7 and 50%, respectively). However, only about one-third of patients are clearly willing to accept admission to intensive care unit, cardiopulmonary resuscitation and mechanical ventilation (36.5, 27.9 and 24%, respectively). A majority of participants (72.1%) preferred to die at home. 18.3% of patients preferred to die at hospital and very few patients (9.6%) accept death in the place determined by the client.

**Table 3:** Preference of patients with cancer pain to end-of-life care (*n* = 104)

Preferences to end of life (EOL) care	N (%)
**Preference to EOL decision-maker**	
Patients’ own	9 (8.7)
Spouse	64 (61.5)
Parents	3 (2.9)
Offspring	24 (23.1)
Relatives	3 (2.9)
Friends	1 (1.0)
**Preference to life-support care**	
**Medication for life-support**	
Yes	62 (59.7)
Not sure	17 (16.3)
No	25 (24.0)
**Intensive care unit**	
Yes	38 (36.5)
Not sure	17 (16.3)
No	49 (47.1)
**Cardiopulmonary resuscitation**	
Yes	29 (27.9)
Not sure	16 (15.4)
No	59 (56.7)
**Nutritional maintain to support life**	
Yes	52 (50.0)
Not sure	19 (18.3)
No	33 (31.7)
**Mechanical ventilation**	
Yes	25 (24.0)
Not sure	20 (19.2)
No	59 (56.7)
**Preferred place of death**	
Home	75 (72.1)
Hospital	19 (18.3)
At the client’s discretion	10 (9.6)

## Discussion

In this cross-sectional study from three coastal provinces in southern China, we found that, (1) EOLD intention of patients with cancer pain was modest with a proportion close to 50%; (2) educational level, history of alcoholism, ECOG score and experience of explosive pain and clinical rescue are independent predictors of willingness to participate in EOLD, and use of a predictive model including above five factors can well discriminate which patients are willing to participate in EOLD; (3) the EOLD maker that a majority of participants preferred was spouse but not their own, only about one-third of patients were clearly willing to accept aggressive life-support therapy, and a majority of participants (72.1%) preferred to die at home.

Currently, advanced care planning (ACP) has been regarded as an effective method in facilitating patient-centered EOL care in the worldwide, which articulates the autonomy of patients’ wishes, values, and goals relevant to their current and future health care [20, 21]. In the Western countries, increasing patients with cancer felt as much involved in EOLD about their future medical treatment and care accordance with their wishes [22]. Previous studies have showed that EOLD consultation are positively associated with receiving appropriate EOL care, increased satisfaction with EOL care, decreased use of hospital care, and increased use of hospice care [20].

On the contrary, very few patients with cancer (2.8 to 18.8%) participated in the process of EOLD in China mainland [14, 15]. Such heavily contrast may be attributed to policy and cultural differences between Western countries and China mainland. In the Western guidelines, patient autonomy is the primary focus of decision-making at the EOL stage [23, 24]. The patients of Western countries tend to express their thoughts on EOLD by a way of ADs, and to believe that this practice is very necessary [25]. However, in China mainland, medical staffs generally discuss the EOL care with the patients’ families but not with the patients’ own. In addition, people’s thoughts are still bound by Chinese traditional culture, especially on the issue of death. Generally, they pay more attention to caring for the elderly and patients than to discussing death related issues with patients or in front of patients [26]. A majority of Chinese peoples don’t understand the content and significance of EOLD. In the face of those patients who are dying, most family members dare not tell the truth of the patient, even oppose telling the patient “bad” diagnosis or prognosis, and choose “concealment”. Simultaneously, medical staffs don’t fully understand the patients’ intention to participate in EOLD due to inadequate communication with patients [27]. All of these may result in patients in China mainland to have little opportunity to understand EOLD related knowledge and to participate in discussion on EOLD, which has been confirmed in recent studies from China mainland [14–16]. More importantly, the true willingness of patients in China mainland to participate in EOLD discussions remains elusive. In this cross-sectional study, 104 patients (46.63%) were willing to participate in EOLD after face-to-face interviews, which was similar as that reported by another study from China [28]. Although this proportion is still lower than that of European and American countries, it is significantly higher than that of our actual participation in EOLD discussions. Thus, the current findings suggest that medical staff in China mainland should communicate and discuss with the patients about EOL care-related topics more proactively, which may lead more patients to participate in EOLD. As for preference to EOL care, our findings were similar to the previous studies from other areas in China mainland [14, 15]. Most participants preferred to their spouse or immediate families as EOLD maker and to die at home. The difference is that we found that about one-third of patients are willing to receive more aggressive life-support treatment (such as intensive care unit, cardiopulmonary resuscitation and mechanical ventilation), which is higher than that in other regions of China mainland (15.5%). This may be explained by different living customs and values in different regions of China [28, 29]. In south China mainland, EOLD will be misunderstood as “giving up treatment and waiting for death passively”. People also have such life value that “our bodies, skin and hair are gifts from our parents”. It is the greatest disrespect to parents if giving up life easily. In fact, similar phenomenon that local living customs and values can affect the patient’s preference to hospice care also exists in the Western countries [30, 31].

In order to provide a high-quality EOL care for cancer pain patients, it is of important clinical significance to identify which patients are more willing to participate in EOLD. So, we further analyzed its related influencing factors. Univariate logistic regression analysis showed that age, educational level, ECOG, history of alcoholism, whether they had experienced explosive pain or clinical rescue, and cause of pain outbreak were the main factors affecting the willingness to participate in EOLD. Although there were significant differences in age and cause of pain outbreak between the patients who willing to participate in EOLD or not, multivariate analysis indicated that both age and cause of pain outbreak are not independent predictors for patients have willingness to participate in EOLD. This was accordance with the results from previous studies [32, 33]. In contrast, independent predictors for patients willing to participate in EOLD were educational level, ECOG, history of alcoholism, whether they had experienced explosive pain or clinical rescue. ECOG score refers to the index of understanding their general health status and treatment tolerance from the patient’s physical strength. The ECOG physical strength score standard is 0–5, the higher the ECOG score, the worse the patient’s health status and the more intolerable to disease-related treatment (for example chemotherapy). The educational level and ECOG score were included into multivariate logistic regression analysis as two continuous variables. The result suggested that those patients with higher educational level or with worse physical condition are more reluctant to participate in EOLD. The willingness to participate in EOLD decreases by 31.7% (95% CI: 0.482–0.966)) for raising each additional educational level and by 35.5% (95% CI: 0.450, 0.925) for increasing each 1-point ECOG, respectively. The results are not similar as a study 5 years ago by Zheng et al. [34]. In that study, they found that higher educational level and higher scores of ECOG were associated with desiring for advanced directives. Such difference may be resulted from the patients included came from different regions of China. In the Zheng’s study, the patients were from west China, while the patients we investigated from coastal regions of southern China. Due to a higher level of economic development in the coastal regions of southern China, people have higher requirements for the quality of life and dying with dignity, which may promote they knew more about EOLD. In addition, the policy related to EOLD in the coastal regions of southern China is also more perfect than that in the western China [35]. Thus, people are more likely to take the initiative to make advance directives and advance care planning at an appropriate time according to their own physical and psychological status, especially for those with higher educational level. Finally, all participants included in this study were patients with cancer pain. Previous study demonstrated that educational level is closely related to whether pain can be controlled well [36]. By comparison with patients with lower educational level, patients with higher educational level have better adherence to the pain management plan recommended by doctors, and sufficient knowledge of pain with believing that pain is just a disease’s concomitant symptom but not necessarily meaning disease deterioration [36, 37]. Under the condition that the pain is well controlled, they usually believe that their disease has not progressed to a life-threatening level and it is not time to consult EOLD with medical staffs. In fact, those patients with higher educational level had lower pain score (supplementary file 2). This may be also one of reasons why they are unwilling to talk about EOLD prematurely. As for physical status, previous study has found that higher ECOG is associated with higher rates of depression or worse psychosocial outcomes, which will lead patients to have more thoughts about suicide or ending their lives as soon as possible instead of peacefully consulting EOLD issues with medical staffs or their families [38, 39]. These results indicate that the caregivers should consult with patients on EOLD at an appropriate time according to their educational attainment and psychological and physical status.

Simultaneously, we also found that a history of alcoholism and whether they had experienced explosive pain or clinical rescue were associated with willing to participate in EOLD. The willingness to participate in EOLD in patients with a history of alcoholism is 8.353-fold higher than those patients without history of alcoholism. The patients who have experienced explosive pain and clinical rescue have stronger willingness to participate in EOLD than those who have not experienced explosive pain and clinical rescue, with 6.367-fold and 3.844-fold higher, respectively. On the one hand, continuous excessive drinking is more easily to damage organs and tissues, perception, cognition, emotion and behavior [40]. Such patients may feel that their disease deteriorated and their physical and psychological status declined faster than non-alcoholics. So, patients with cancer pain who have a history of alcoholism are more willing to accept hospice decision-making early. On the other hand, unfortunate clinical experience will attack the patients’ confidence in disease recovery. Explosive pain cannot be generally relieved by using prescription of opioids and has been seen as an important cause that cancer patients may seek to hasten their deaths [41]. The patients are aware of ineffective treatment and worry about sudden deterioration of condition so that they have no opportunity to make EOLD. Thus, such patients are more likely to make EOLD together with medical staff and family members, which is similar as the finding from the Research Report of Chinese nursing homes in Hong Kong [42]. Finally, we established a predicting model for willing to participate in EOLD combined above five factors, which showed a good discrimination (area under receiver operating characteristic curve: 0.849, 95% CI: 0.796–0.899, *P* <  0.001) and calibration (Hosmer-Lemeshow Test: Chi-square = 10.103, *P* = 0.258) for which patients more willing to participate in EOLD. Thus, such model may be helpful for us to improve patients’ knowledge of EOLD and to provide EOL care in line with patients’ wishes in the clinical practice. Of course, it is undeniable that our study also has certain limitations. First, only few hospitals were included in the survey, which may lead to the result bias. Second, our results only reflect the patients’ willingness at the time of the survey. The patient’s attitude towards whether to participate in EOLD consultation may be shifted over time or with his own health status. Finally, we did not further analyze the reasons why some patients were not sure whether to participate in EOLD. Therefore, it is required to conduct a large-sample longitudinal study addressing on these issues above in the future.

## Conclusions

Our findings suggest that the willingness of patients with cancer pain to participate in EOLD is at a modest level in three coastal provinces of southern China. Some factors including demographic and clinical characteristics influenced such willingness. This study highlights the importance of considering the above factors when making EOL care plans for patients with cancer pain, which will be helpful to improve their quality of life in the EOL stage.

## Supplementary Information

**Additional file 1.**

## Data Availability

The Dataset used and/or analyzed in the current study available from corresponding author on reasonable request.

## Abbreviations

ACP: advanced care planning
ADs: advance directives
BMI: body mass index
ECOG: Eastern Cooperative Oncology Group
EOL: end of life
EOLD: end of life decision
NRS: numerical rating scale
ROC: receiver operating characteristic curve
