# Supplementary material for: Willingness of patients with cancer pain to participate in end-of-life decisions: a multi-center cross-sectional study from three coastal provinces in southern China
Source: BMC Palliat Care. 2022 Nov 26;21:207. doi: 10.1186/s12904-022-01108-x (PMC9700943; doi:10.1186/s12904-022-01108-x)
Supplement: Supplementary file 1 — Additional file 1. [file 12904_2022_1108_MOESM1_ESM.docx]

**Supplementary files**

**1. Survey on willingness of cancer pain patients to participate in end-of-life decision-making (EOLD)**

Hello, in order to explore the willingness of cancer pain patients to participate in EOLD and potential influencing factors, we sincerely invite you take part in this survey. Your information will be confidential. Thank you for your valuable time and your cooperation!

| **Part I. Demographic information** | | | |
| --- | --- | --- | --- |
| Bed No.：___________ | Age：__________ | | Gender：__________ |
| Admission ID：_________ | Diagnosi：___________ | | Religiosity：◎Yes ◎No |
| Height：_____cm，Weight：_____Kg；  BMI：__________Kg/m^2^ | | Residence：◎ Rural ◎Urban | |
| Marital status ：◎ Unmarried ◎ Married ◎Divorced/Widowed | | | |
| Educational level：  ◎Primary school/below ◎Middle school ◎ High school ◎ Above high school | | | |
| House-held income (CNY per Capita each month):  ◎ Low（<2000 ￥） ◎Middle（2000-5000 ￥） ◎ High（≥5000 ￥） | | | |
| Previous smoking：  ◎Yes ◎No | | Previous alcoholism：  ◎Yes ◎No | |
| **Part II. Disease-related information** | | | |
| Tumor site ：  ◎ Digestive ◎ Respiratory ◎ Urinary ◎ Gynecological ◎ Other | | | |
| Pathological stage：◎Ⅰ ◎Ⅱ ◎Ⅲ ◎Ⅳ | | | |
| What kind of painkiller you use currently：______________；  Daily dose：______； analgesic ：____； Frequency of administration：__________； | | | |
| Which ladder the analgesic is：◎First ◎Second ◎Third | | | |

| **Part III. Physical performance** |
| --- |
| 1. Please select the most appropriate pain score for you: (0 is painless, 10 is the most painful and Intolerable):  0 1 2 3 4 5 6 7 8 9 10 |
| 2. Which of the following activity status do you currently meet best:  ◎ 0 Fully active, able to carry on all pre-disease performance without restriction.  ◎ 1 Restricted in physically strenuous activity but ambulatory and able to carry out work of a light or sedentary nature, e.g., light house work, office work.  ◎ 2 Ambulatory and capable of all selfcare but unable to carry out any work activities; up and about more than 50% of waking hours.  ◎ 3 Capable of only limited selfcare; confined to bed or chair more than 50% of waking hours.  ◎ 4 Completely disabled; cannot carry on any selfcare; totally confined to bed or chair.  ◎ 5 Dead. |
| 3. Please select the most appropriate score for nutritional status according to NRS 2002:  ◎ 0 ◎1 ◎2 ◎3 ◎4 ◎5 ◎6  Age if ≥ 70 years: add 1 to total score above =age-adjusted total score: ◎ Yes ◎ No |
| **Part IV. Willingness to participate in EOLD and Preferences to end of life (EOL) care** |
| **Part IV-A. Willingness to participate in EOLD** |
| 1. Would you like to take part in the consultation for EOLD?  ◎Yes ◎Not sure ◎No |
| 2. Did you experience explosive pain during the treatment (Sudden unbearable pain with needing medical help)?  ◎Yes ◎No |
| 3. What do you think is the cause for the outbreak of pain?  ◎Disease deterioration ◎Improper use of analgesics ◎Other |
| 4. Have you ever experienced a clinical rescue when your life is in danger?  ◎Yes ◎No |
| Only if you are willing to participate in EOLD, please continue to complete the following questions in Part IV-B. |
| **Part IV-B. Preferences to end of life (EOL) care** |
| 1. Who do you prefer to make the final decision for you when you are dying?  ◎ Yourself ◎Spouse ◎Parents ◎Offspring ◎Relatives ◎Friends |
| 2. Are you willing to accept the support-life medication when your life is in danger?  ◎Yes ◎Not sure ◎No |
| 3. Are you willing to stay in the intensive care unit for further treatment when you are in critical condition?  ◎Yes ◎Not sure ◎No |
| 4. Are you willing to accept cardiopulmonary resuscitation (CPR) to save your life when you are in danger when your life is in danger?  ◎Yes ◎Not sure ◎No 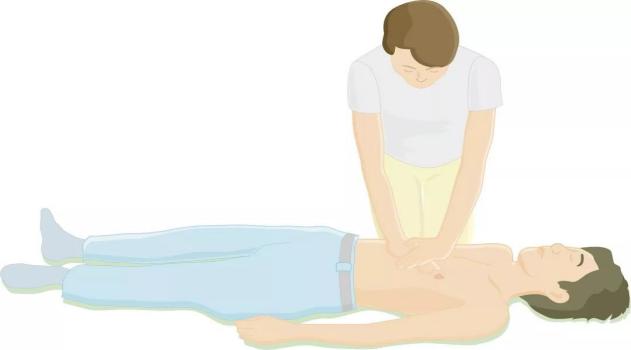 |
| 5. Are you willing to accept mechanical ventilation to prolong your life when you are in danger when your life is in danger?  ◎Yes ◎Not sure ◎No 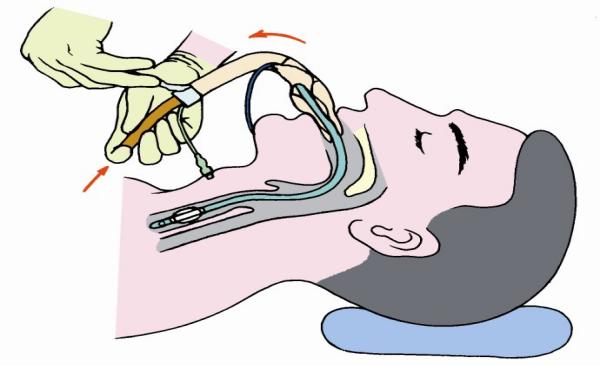 |
| 6. Are you willing to accept nutritional maintain to support life when you are in danger when your life is in danger?  ◎Yes ◎Not sure ◎No |
| 7. Where is your preferred place of death when your life is irreversible?  ◎Home ◎Hospital ◎At the client's discretion |

**2. Difference in pain score (number scale rating, NRS) among patients with different educational level**

NRS was presented as mean and standard deviation, and compared by using one-way ANOVA Test and Bonferroni T test. Correlation between educational level and NRS was analyzed by using Pearson test. All P values were two-tailed, and statistical significance was set at P <0.05. The results showed in supplementary figure 1. The NRS was 2.53 ± 0.91 in the group primary school and below, 2.42 ± 1.12 in the group middle school, 1.95 ± 0.85 in the group high school, and 1.81 ± 0.99 in the group above high school, respectively. The NRS in the group high school and group above high school were significantly higher than that in the group primary school and below (P < 0.05) .The NRS in the group middle school was lower than that of group high school (P = 0.085) and group above high school (P = 0.035). There was no significant difference between group primary school and below and group middle school and between group high school and group above high school. However, there was a trend that the higher educational level, the smaller the NRS is, despite of no significant difference in NRS between group primary school and below and group middle school and between group high school and group above high school. There was a significant negative correlation between the educational level and NRS (correlation coefficient = -0.268, P < 0.001).


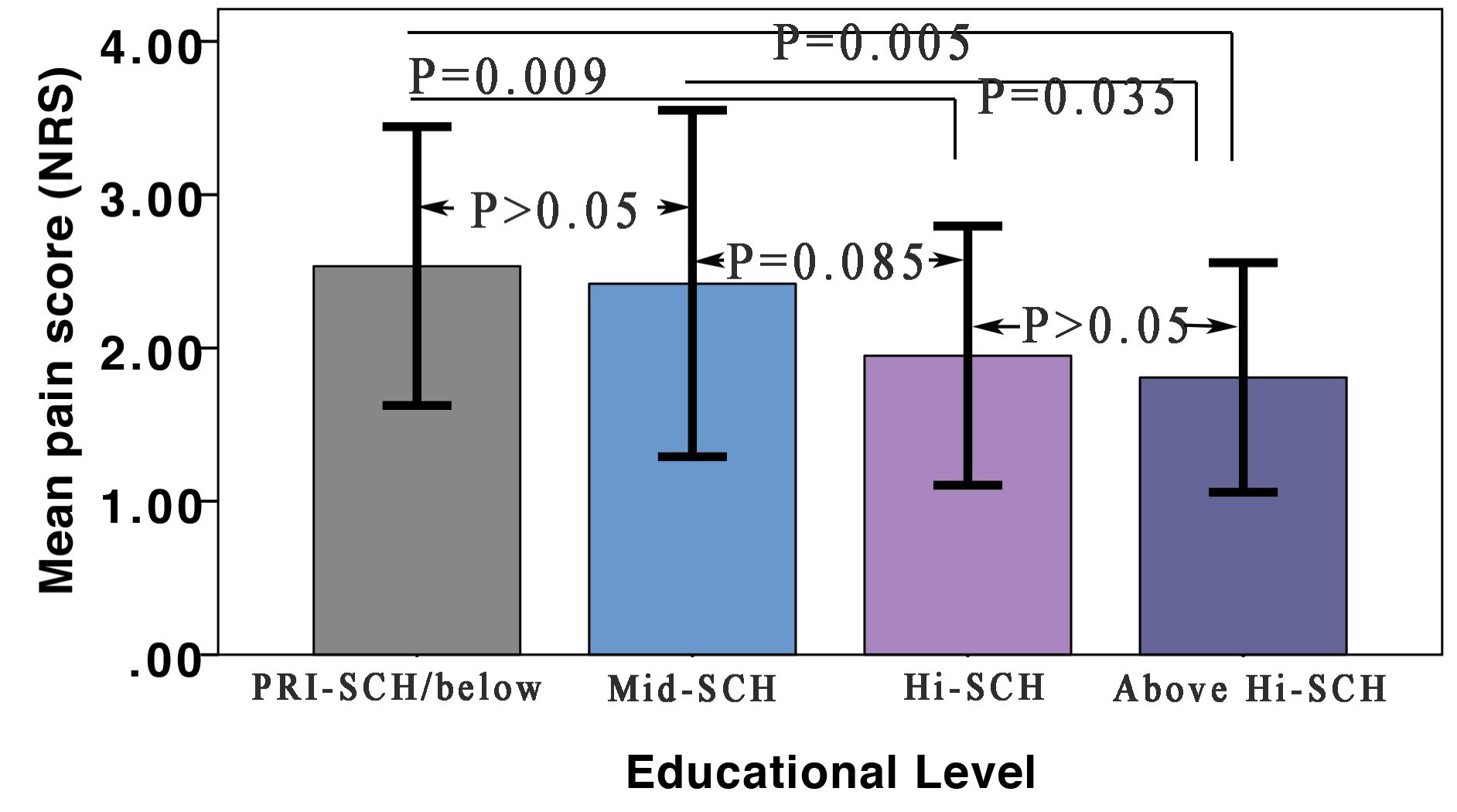


Supplementary figure 1. The NRS in patients with different educational level. PRI-SCH: primary school; Mid-SCH: middle school; Hi-SCH: high school.
